# The Fault Tolerant Control Design of an Intensified Heat-Exchanger/Reactor Using a Two-Layer, Multiple-Model Structure †

**DOI:** 10.3390/s20174888

**Published:** 2020-08-28

**Authors:** Menglin He, Zetao Li, Boutaib Dahhou, Michel Cabassud

**Affiliations:** 1 Electrical Engineering College, Guizhou University, Guiyang 550025, China; menglin.he@laas.fr; 2 Key Laboratory for Internet plus Smart Manufacture of Guizhou Province, Guiyang 550025, China; 3 LAAS-CNRS, Université de Toulouse, CNRS, INSA, UPS, 31400 Toulouse, France; boutaib.dahhou@laas.fr; 4 LGC, Université de Toulouse, CNRS/INPT/UPS, 31432 Toulouse, France; michel.cabassud@ensiacet.fr

**Keywords:** fault tolerant control, heat-exchanger/reactor, multiple model

## Abstract

The heat-exchanger/reactor (HEX reactor) is a kind of plug-flow chemical reactor which combines high heat transfer ability with good chemical performances. It was designed under the popular trend of process intensification in chemical engineering. Previous studies have investigated its characteristics and developed its nominal model. This paper is concerned with its fault tolerant control (FTC) applications. To avoid the difficulties and nonlinearities of this HEX reactor under chemical reactions, a two-layer, multiple-model structure is proposed for designing the FTC scheme. The first layer focuses on representing the nonlinear system with a bank of local linear models while the second layer uses model banks for approaching faulty situations. Model banks are achieved by system identification, and the corresponding controller banks are designed using model predictive control (MPC). The unscented Kalman filter (UKF) is introduced to estimate the states and form the fault detection and isolation (FDI) section. Finally, the FTC simulation and validation results are presented. The idea of a two-layer, multiple-model structure presents a general framework for FTC design of complex and highly nonlinear systems, such as the HEX reactor, whose mathematical model has been created. It implements the design process in an unusual way and is also worth trying on other cases.

## 1. Introduction

Recently, process intensification [1,2,3], which aims at replacing the traditional batch reactors with novel ones by combining two or more traditional operations in a hybrid unit, is getting more and more popular. The heat-exchanger/reactors mentioned in this paper fall under this trend of process intensification. The heat-exchanger (HEX) reactors are well-known for their thermal and hydrodynamic performances [4], and they are also widely studied for highly exothermic reactions [5]. Characteristics and mathematical models of the HEX reactor have been investigated before. This paper focuses on its fault tolerant control design.

As is known to all, automatic facilities are used widely and are also getting advanced and complicated. Developing security schemes for them is always a demanding task. Among all the techniques, fault tolerant control (FTC) receives more and more attention because it can guarantee the control performance in faulty situations [6,7]. Generally, FTC strategies are classified into active and passive ones [8]. Passive FTC strategies perform more like robust control, which could be pre-designed and run without the need for either real-time fault detection and diagnosis (FDD) or control reconfiguration [9]. Active FTC, on the other hand, automatically adjusts the control law using the information given by a fault detection and isolation module. Additionally, it tries to satisfy the control objectives with minimum performance degradation [10] after the fault’s occurrence. The active FTC approach is more flexible when dealing with different types of faults, while the passive approach is easy to implement since it does not need an FDD unit or a reconfiguration mechanism [11].

Among these active FTC approaches, studies on multiple-model based reconfigurable control have drawn increasing attention [12]. The idea of multiple-model approach was originally proposed in [13] and is systematically described in [14]. Due to the development of computing devices, doing parallel calculations of multiple models is not longer a problem to hardware; that change intensively boosted the growth of the multiple-model approach. It is not only used for controller design (see [15,16]) but is also applied in the domain of system reliability for things such as fault diagnosis and fault tolerant control; see [17,18]. Multiple model approaches deal with fault diagnosis problems in a way to avoid the complicated process of observer and controller design of the real system. However, complexities still exist in integrated controller design for sub-models, especially when the considered system is complex and highly nonlinear.

This paper uses a strategy which combines a model-based method and a data-driven method to finish the job of FTC design for the HEX reactor. During this process, the multiple-model approach is applied in two dimensions to form a two-layer, multiple-model structure for the precise system representation and FTC strategy implementation. The construction of the multiple-model banks utilizes a system identification approach. Model predictive control is applied in each sub-controller using parameters of the two-layer multiple models. Before implementing the controller banks, adjustments toward them are done in the second layer to unify the performances. To monitor the real plant and have it give out its fault information for the following control compensation, an fault detection and isolation (FDI) section was designed using the unscented Kalman filters. Simulations under the assumptions of heat transfer coefficient faults and input utility temperature faults were carried out to show the performance of the proposed FTC strategy for the HEX reactor.

This paper is organized as follows. Section 2 introduces the targeting HEX reactor. Section 3 constructs the two-layer model banks of the HEX reactor step by step. Section 4 presents the model predictive control (MPC)-based controller design and tuning. Section 5 states the FDI design and FTC strategies and gives out the simulation results with discussions. In the last section, a conclusion of this paper is given.

## 2. Modeling and Problem Statement

**Figure 1:**
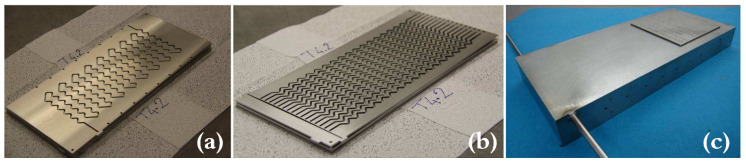
(**a**) Process plate; (**b**) utility plate; (**c**) the HEX reactor after assembly [20].

The HEX reactor is designed under the concept of a plate heat exchanger in a module. As is shown in Figure 1, there are two kinds of plates which build up the targeting HEX reactor of this paper, namely, the process plate and the utility plate. Chemical reactions would take place in process channels while utility fluids would be injected into utility channels to bring in or take away heat. Detailed parameters can be found in our previous research [19].

When modeling this reactor, channels, which are engraved in the thin metal plate, are virtually considered to be an independent plate, leaving the metal plate to be a kind of component called plate wall. Thus, we have three types of components: process channels, utility channels and plate wall. The reactor is then represented by a series of perfectly stirred tank reactors (called cells). In this way, the flow modeling method [21] could be introduced in the modeling part. To investigate the characteristics of the HEX reactor, the reaction of sodium thiosulfate oxidation with hydrogen peroxide, which is a strong exothermic reaction, is introduced both in the experiments and modeling sections.

Chemical equation of this reaction is:

(1) $$2{\mathrm{Na}}_{2}S_{2}O_{3}+4H_{2}O_{2}\to {\mathrm{Na}}_{2}S_{3}O_{6}+{\mathrm{Na}}_{2}SO_{4}+4H_{2}O$$

Thus, dynamics of the HEX reactor with the reaction can be given [20]:

(2) $$\left\{\begin{array}{cc} {\dot{T}}_{p} & =\frac{F_{p1}+F_{p2}}{V_{p}}\left(T_{pin}-T_{p}\right)+\frac{h_{p}A_{p}}{\rho_{p}V_{p}C_{p}}\left(T_{w}-T_{p}\right)+\frac{\Delta H}{\rho_{p}C_{p}}k_{j}^{0}exp\left(-\frac{E_{a}}{R(T_{p}+273.15)}\right)C_{1}C_{2}\\ {\dot{T}}_{u} & =\frac{F_{u}}{V_{u}}\left(T_{uin}-T_{u}\right)+\frac{h_{u}A_{u}}{\rho_{u}V_{u}C_{u}}\left(T_{w}-T_{u}\right)\\ {\dot{T}}_{w} & =\frac{h_{p}A_{p}}{\rho_{w}V_{w}C_{w}}\left(T_{p}-T_{w}\right)+\frac{h_{u}A_{u}}{\rho_{w}V_{w}C_{w}}\left(T_{u}-T_{w}\right)\\ {\dot{C}}_{1} & =\frac{F_{p1}+F_{p2}}{V_{p}}\left(C_{1in}-C_{1}\right)-2k_{j}^{0}exp\left(-\frac{E_{a}}{R(T_{p}+273.15)}\right)C_{1}C_{2}\\ {\dot{C}}_{2} & =\frac{F_{p1}+F_{p2}}{V_{p}}\left(C_{2in}-C_{2}\right)-4k_{j}^{0}exp\left(-\frac{E_{a}}{R(T_{p}+273.15)}\right)C_{1}C_{2} \end{array}\right.$$

where $T_{p}$, $T_{u}$, $T_{w}$, $C_{1}$, $C_{2}$ are temperature of the process channel, temperature of the utility channel, temperature of the plate-wall, concentration of ${\mathrm{Na}}_{2}S_{2}O_{3}$, and concentration of $H_{2}O_{2}$ respectively. $F_{p1}$, $F_{p2}$ and $F_{u}$ are the input flow rate of process and utility channels. *V*, *A*, *h*, ρ and *C* stand for volume, heat exchange area, heat transfer coefficient, density and specific heat capacity. $k_{j}^{0}$ is a pre-exponential factor of the reaction; $E_{a}$ is the activation energy; *R* is the perfect gas constant and ΔH is the unit heat generated by the reaction. Detailed values of these parameters can be found in [20].

Apparently, studies on fault detection, isolation and identification for the HEX reactor are the prerequisite for further implementations. An FTC system is able to recover and continue to operate as in normal conditions or to maintain the stability to the desired level when a fault occurs. Developing suitable FTC strategies becomes a must to ensure the reliability.

For simplicity, we set the flow-rate of utility fluid $F_{u}$ as the only input and the temperature of process channel $T_{p}$ as the only output of the system to start from a SISO case. This hypothesis is consistent with the reality that the inputs of reactants would generally have a fixed optimal proportion, while no restrictions would be set on utility flow-rate. As for the output, the temperature of the reactants is always an important index of the reaction. Thus, $F_{u}$ and $T_{p}$ in (2) are suitable to set as the input and output of the system.

## 3. Two-Layer Multiple Model Structure Construction

### 3.1. The First Layer of the Multiple Model Structure

The two-layer, multiple-model structure proposed here is generally an expansion of the classical multiple-model approach. As is known, multiple-model approaches use the divide-and-conquer strategy to deal with complexity in engineering systems [14]. For a complex nonlinear system, local models, which are valid for certain ranges of workspace, are combined to describe the complete workspace.

Since the original nonlinear model (2) is available, virtual experiments could be done by simulations to generate enough data for local model identification. As the HEX reactor is considered as a SISO system first, the input $F_{u}$ could be a suitable candidate of decision variable [22] which indicates the validity of local models.

**Figure 2:**
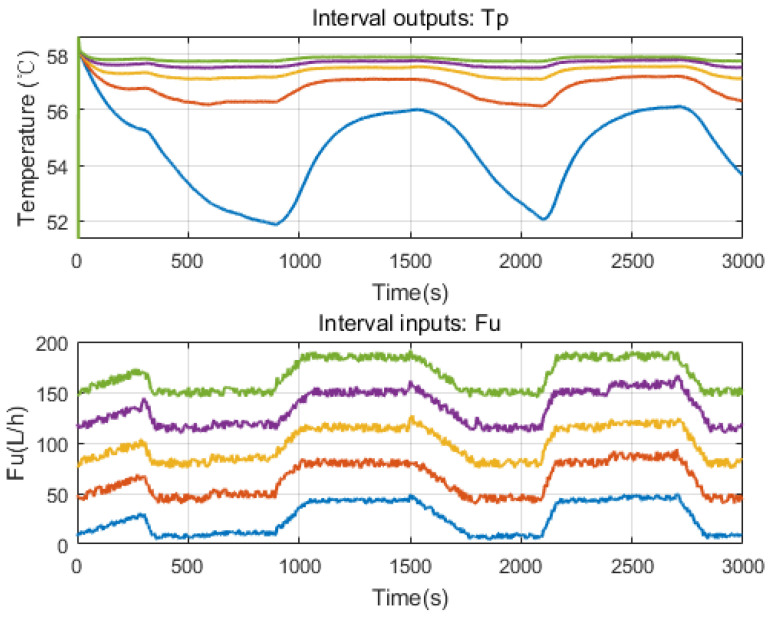
The interval inputs and outputs from virtual experiments (the color indicates the IO pair).

The first layer of multiple models is then created using the system identification method. For the given HEX reactor, assume that the input $F_{u}$ ranges from 0 to 200 L/h. First, interval inputs could be generated by adding white noise to base signals (see Figure 2: Fu).

By applying the interval inputs to model (2) one by one, corresponding outputs could be generated (see Figure 2: Tp). Thus, several sets of IO data are prepared and we come to the second step: local model identification. The following ARX structure is chosen for local models.

(3) $$x_{j}\left(k+1\right)=a_{1j}x_{j}\left(k\right)+a_{2j}x_{j}\left(k-1\right)\cdots +b_{1j}u\left(k-d\right)+b_{2j}u\left(k-d-1\right)\cdots +c_{j}$$

where *j* denotes the number of local models; $a_{ij}$, $b_{ij}$ are parameters of the regressors; $c_{j}$ is an offset; and *d* is the time delay.

After investigating, the residence time of process fluid would be a key parameter for estimating the time delay. In this work, we suppose that $F_{p1}$ and $F_{p2}$ are equal to 4.7 L/h and 2.3 L/h respectively. The residence time is then around 10 s. Thus, the time delay is 2 steps when sample time is set to 5 s. Orders of the local model could be found using a modified Lipschitz-quotient method proposed in [23].

When local models are identified by the classical least square approach, they are combined by a switching function to generate an overall output according to current input. A multiple-model bank in the first layer is given by:

(4) $$\left\{\begin{array}{l} x_{j}\left(k+1\right)=a_{1j}x_{j}\left(k\right)+a_{2j}x_{j}\left(k-1\right)\cdots +b_{1j}u\left(k-d\right)+b_{2j}u\left(k-d-1\right)\cdots +c_{j}\\ y\left(k\right)=f(u\left(k\right),x_{j}\left(k\right)) \end{array}\right.$$

where *y* is the overall estimation of $T_{p}$ given by the model bank; *f* is a switching function whose decision variable and candidate outputs are the input *u* and outputs of local models $x_{j}$ respectively; *u* is $F_{u}$ in (2).

**Figure 3:**
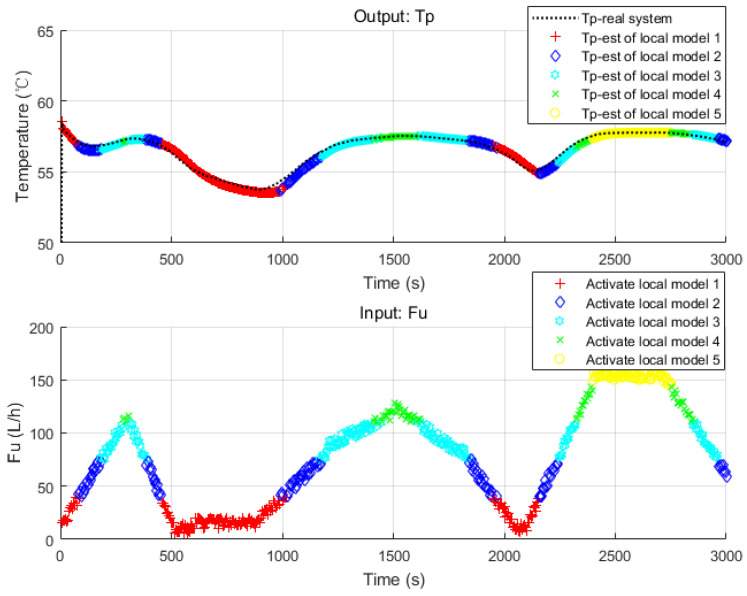
Verification of the first layer model bank using inputs of integral range.

For verifying the accuracy, a set of input signals, which vibrates in a wide range, is sent to both the original nonlinear model and the first layer model bank (see Figure 3).

**Figure 4:**
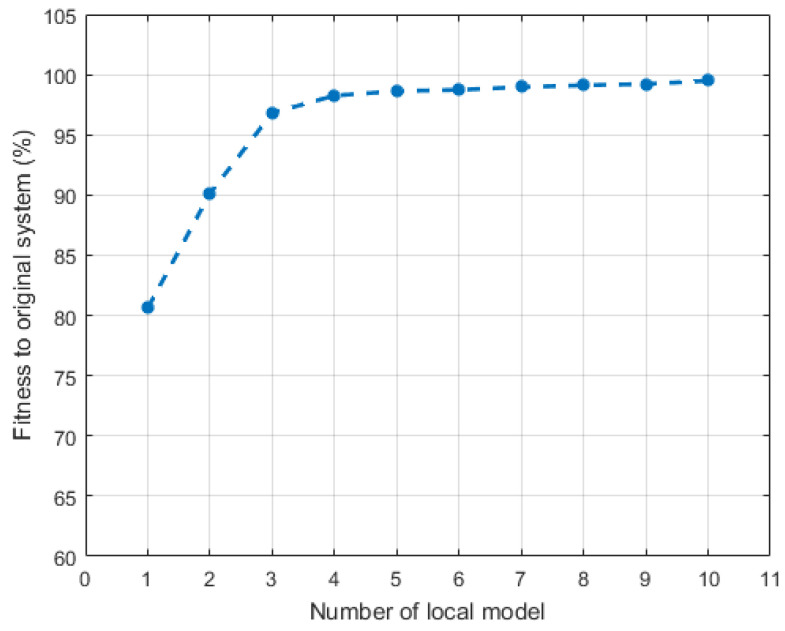
The accuracies of the multiple-model bank with different total numbers of local models.

According to Figure 3, the behavior of the nonlinear system is well captured by the model bank with five local models. It also shows that the switching strategy is used and a different local model is activated when input $F_{u}$ goes into its corresponding interval. The number of local models is a parameter which should be investigated. Figure 4 shows the accuracies of model banks with different quantities of local models. Apparently, for the case of our HEX reactor, five local models are enough to constitute a model bank to describe the original system in a highly economical and accurate way.

### 3.2. Construction of the Second Layer Model Bank

**Figure 5:**
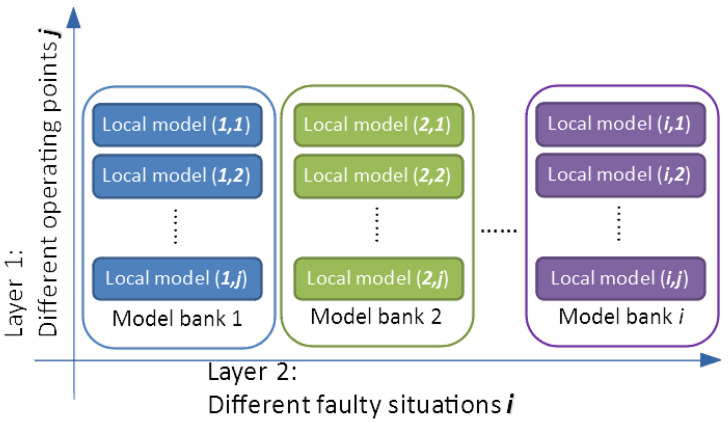
Two-layer multiple models.

The construction of the second layer, which concerns faults, is a simple extension of the same steps to the second dimension. In this paper, we mainly focus on dynamic faults, i.e., the changes of plant parameters. For simplicity, a single fault is considered here. Thus, as defined in biographies, a fault would be caused by the deviation of a parameter from its nominal value [6]. When the reactor works, there is a possibility that materials in the fluids may stay at the inner surfaces of the channels, which would affect the performance of the heat exchange process. It is a typical fault of this reactor and could be considered as the change of heat transfer coefficient *h*. Therefore, the value of this parameter is chosen to set faulty intervals for the construction of the second layer model banks. Virtual experiments were carried out to generate IO data with these intervals. We set four faulty situations (80%h, 60%h, 40%h, 20%h) along with one nominal case (100%h). By repeating the identification process of the first layer model bank, a two-dimension multiple-model matrix was given (see Figure 5).

## 4. Two-Layer Controller Bank Design

Controller design for the complex HEX reactor is easy now because the highly nonlinear system is described by equivalent model banks using linear local models. The task becomes designing controllers for these homogeneous local linear models where nearly all kinds of controllers can be competent. Thus, several controller banks, which are considered for the second layer, are constructed according to the model banks. Inside each controller bank, multiple controllers are defined as in the first layer.

### 4.1. Controller Bank Design

Model predictive control [24,25], for its popularity and capability of handling hard constraints in the process control domain, was chosen for constructing the corresponding controller banks. To achieve that, some transformations should be done on the local models. First, we transform them from ARX (3) to state-space-like form by defining a new state vector and input vector in the following way:

(5) $$x_{mj}\left(k\right)=\left[\begin{array}{c} x_{j}\left(k\right)\\ x_{j}\left(k-1\right)\\ \vdots \end{array}\right]$$

(6) $$u_{j}\left(k\right)=\left[\begin{array}{c} u(k-d)\\ u(k-d-1)\\ \vdots \end{array}\right]$$

where u(k) and $x_{j}\left(k\right)$ stand for the input and the local estimation of state at step *k*. The lengths of the two vectors are dependent on the order and time delay of the local model.

In that way, the following state-space-like model is given:

(7) $$\left\{\begin{array}{l} \left[\begin{array}{c} x_{j}\left(k+1\right)\\ x_{j}\left(k\right)\\ \vdots \end{array}\right]=A_{mj}\left[\begin{array}{c} x_{j}\left(k\right)\\ x_{j}\left(k-1\right)\\ \vdots \end{array}\right]+Bmj\left[\begin{array}{c} u(k-d)\\ u(k-d-1)\\ \vdots \end{array}\right]+\left[\begin{array}{c} c_{j}\\ 0\\ \vdots \end{array}\right]\\ {\hat{y}}_{j}\left(k\right)=\left[\begin{array}{ccc} 1 & 0 & \cdots \end{array}\right]\left[\begin{array}{c} x_{j}\left(k\right)\\ x_{j}\left(k-1\right)\\ \vdots \end{array}\right] \end{array}\right.$$

where $A_{m}$ and $B_{m}$ are matrices calculated from the transformation of the ARX model and the item containing $c_{j}$ concerns about the offset in (3). Model (7) could be written in (8) for short:

(8) $$\left\{\begin{array}{l} x_{mj}\left(k+1\right)=A_{mj}x_{mj}\left(k\right)+B_{mj}u_{j}\left(k\right)+c_{j}\\ {\hat{y}}_{j}\left(k\right)=C_{mj}x_{mj}\left(k\right) \end{array}\right.$$

By making a difference on state and input vectors, offset vector $c_{j}$ could be eliminated:

(9) $$\Delta x_{mj}\left(k+1\right)=x_{mj}\left(k+1\right)-x_{mj}\left(k\right)$$

(10) $$\Delta u_{j}\left(k\right)=u_{j}\left(k\right)-u_{j}\left(k-1\right)$$

Define a new state vector:

(11) $$x_{j}\left(k\right)={\left[\begin{array}{cc} \Delta x_{mj}\left(k\right) & {\hat{y}}_{j}\left(k\right) \end{array}\right]}^{T}$$

Then, an augmented system is given by combining (7)–(10):

(12) $$\left\{\begin{array}{l} x_{j}\left(k+1\right)=\left[\begin{array}{cc} A_{mj} & o\\ C_{mj}A_{mj} & 1 \end{array}\right]\left[\begin{array}{c} \Delta x_{mj}\left(k\right)\\ {\hat{y}}_{j}\left(k\right) \end{array}\right]+\left[\begin{array}{c} B_{mj}\\ C_{mj}B_{mj} \end{array}\right]\Delta u_{j}\left(k\right)\\ {\hat{y}}_{j}\left(k\right)=\left[\begin{array}{cc} o & 1 \end{array}\right]\left[\begin{array}{c} \Delta x_{mj}\left(k\right)\\ {\hat{y}}_{j}\left(k\right) \end{array}\right] \end{array}\right.$$

Additionally, (12) is written in (13) for short:

(13) $$\left\{\begin{array}{l} x_{j}\left(k+1\right)=A_{j}x_{j}\left(k\right)+B_{j}\Delta u_{j}\left(k\right)\\ {\hat{y}}_{j}\left(k\right)=C_{j}x_{j}\left(k\right) \end{array}\right.$$

Therefore, a standard MPC design [24] is carried out in the following steps based on (13). First, we assume that the future control signal is known. Then, the future states and outputs are predicted according to current data in step *k*:

(14) $$Y_{j}\left(k\right)=F_{j}X_{j}\left(k\right)+\phi_{j}\Delta U_{j}$$

where $Y_{j}\left(k\right)$ and $X_{j}\left(k\right)$ are predictions of states and outputs computed at step *k*; $\Delta U_{j}$ is the future incremental control input. Elements in (14) are constructed in the following way:

(15) $$Y_{j}\left(k\right)=\left[\begin{array}{c} {\hat{y}}_{j}\left(k+1|k\right)\\ {\hat{y}}_{j}\left(k+2|k\right)\\ \vdots \\ {\hat{y}}_{j}\left(k+N_{p}|k\right) \end{array}\right]$$

(16) $$X_{j}\left(k\right)=\left[\begin{array}{c} x_{j}\left(k+1|k\right)\\ x_{j}\left(k+2|k\right)\\ \vdots \\ x_{j}\left(k+N_{p}|k\right) \end{array}\right]$$

(17) $$\Delta U_{j}=\left[\begin{array}{c} \Delta u_{j}\left(k\right)\\ \Delta u_{j}\left(k+1\right)\\ \vdots \\ \Delta u_{j}\left(k+N_{c}-1\right) \end{array}\right]$$

(18) $$F_{j}=\left[\begin{array}{c} C_{j}A_{j}\\ C_{j}A_{j}^{2}\\ \vdots \\ C_{j}A_{j}^{N_{p}} \end{array}\right]$$

(19) $$\Phi_{j}=\left[\begin{array}{cccc} C_{j}B_{j} & 0 & \cdots & 0\\ C_{j}A_{j}B_{j} & C_{j}B_{j} & \cdots & 0\\ \vdots & \vdots & \ddots & \vdots \\ C_{j}A_{j}^{N_{p}-1}B_{j} & C_{j}A_{j}^{N_{p}-2}B_{j} & \cdots & C_{j}A_{j}^{N_{p}-N_{c}}B_{j} \end{array}\right]$$

where $N_{p}$ and $N_{c}$ are prediction horizon and control horizon respectively.

For a given reference signal $R_{s}$, prediction error can be defined:

(20) $$E_{j}=R_{s}-Y_{j}$$

The following cost function is given based on the prediction error:

(21) $$J_{j}=E_{j}^{T}E_{j}+\Delta U_{j}^{T}\bar{R}\Delta U_{j}$$

where $\bar{R}$ is a positive penalty parameter concerning about the magnitude of control input.

By letting the first derivative of $J_{j}$ (22) be equal to zero, the optimal control value (23) can be calculated:

(22) $$\frac{\partial J_{j}}{\partial \Delta U_{j}}=-2\Phi_{j}^{T}\left(R_{s}-F_{j}X_{j}\left(k\right)\right)+2\left(\Phi_{j}^{T}\Phi_{j}+\bar{R}\right)\Delta U_{j}$$

(23) $$\Delta U_{j}={\left(\Phi_{j}^{T}\Phi_{j}+\bar{R}\right)}^{-1}\Phi_{j}^{T}\left(R_{s}-F_{j}X_{j}\left(k\right)\right)$$

For each calculation step, only the first element of $\Delta U_{j}$ will be implemented. Calculations would be done again for the next step to carry out the dynamic optimization strategy of MPC.

All local controllers could be created in the same way according to their corresponding local models. A similar switching strategy using input as the decision variable is implemented to manage the controllers to give out an overall output of the controller bank. In this way, controller design of the complex nonlinear system is solved by designing sub-controllers for simple linear local models. Controller design for the second layer is carried out the same way using the information of the second layer model banks.

### 4.2. Tuning of the Second Layer Controller Banks

The key problem is that the performances of all the controller banks should be tuned to be similar. Only that way can the FTC strategy behave well when the controller bank is switched during faulty situations.

**Figure 6:**
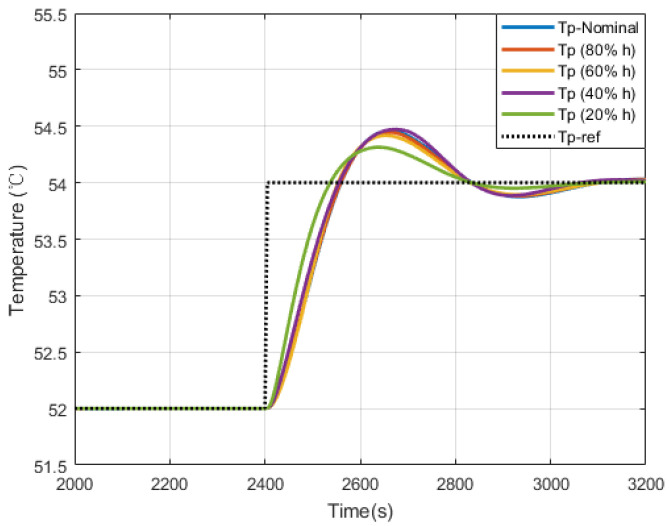
Performances of controller banks under the same reference Tp-refafter tuning.

Among the three parameters which could be adjusted in MPC, penalty $\bar{R}$ is the most sensitive one. After setting a standard performance in the nominal controller bank, other controller banks could achieve similar performances by adjusting $\bar{R}$. Here we choose the convergence time as the index, and introduce binary search to finish the job to have a result as shown in Figure 6. The corresponding vector for $\bar{R}$ of each controller bank in Figure 6 is $\left[\begin{array}{ccccc} 0.0300 & 0.0149 & 0.1453 & 0.0755 & 0.0093 \end{array}\right]$.

## 5. FTC Implementation and Simulations

### 5.1. UKF Based FDI Strategy

The unscented Kalman filter was proposed by Julier and Uhlman in the context of state-estimation for nonlinear systems [26]. To avoid the linearization process in the famous extended Kalman filter (EKF), a finite set of weighed sigma points will be generated by the UKF to compute the predicted states and measurements and the associated covariance matrices [27]. Generally, the UKF estimates the states of nonlinear systems according the flowing steps.

Step 1: Determine the set of sigma points and calculate the corresponding weights.

(24) $${\hat{x}}_{k-1|k-1}^{a}=\left[\begin{array}{c} {\hat{x}}_{k-1|k-1}\\ 0\\ 0 \end{array}\right]$$

(25) $$P_{k-1|k-1}^{a}=\left[\begin{array}{cccc} \; & P_{k-1|k-1} & 0^{L\times q} & 0^{L\times r}\\ \; & 0 & Q_{k-1} & 0\\ \; & 0 & 0 & R_{k-1} \end{array}\right]$$

(26) $$\chi_{k-1}^{a}={\hat{X}}_{k-1|k-1}^{a}+\left[\begin{array}{cccc} \; & 0 & \sqrt{\left(L^{a}+\lambda \right)P_{k-1|k-1}^{a}} & -\sqrt{\left(L^{a}+\lambda \right)P_{k-1|k-1}^{a}} \end{array}\right]$$

(27) $$W_{i}=\left\{\begin{array}{ll} \frac{\lambda }{2(L^{a}+\lambda )}, & i=1\\ \frac{1}{2(L^{a}+\lambda )}, & otherwise \end{array}\right.$$

where *L* is the dimension of state vector of the original system, *Q* and *R* are tuning parameters of the filter, λ is the scaling factor denoting the distance for choosing sigma points and *W* is the weight.

Step 2: Prediction.

(28) $$\chi_{k}^{x}=f\left(\chi_{k-1}^{x},\chi_{k-1}^{w},u_{k-1}\right)$$

(29) $$\gamma_{k}=h\left(\chi_{k}^{x},\chi_{k-1}^{v},u_{k}\right)$$

(30) $${\hat{x}}_{k|k-1}=\sum_{i=1}^{2L^{a}+1}W_{i}\chi_{i,k}^{x}$$

(31) $${\hat{y}}_{k}=\sum_{i=1}^{2L^{a}+1}W_{i}\gamma_{i,k}$$

where f(·) and h(·) are the nonlinear system function and output function respectively.

Step 3: Update.

(32) $$P_{k|k-1}=\sum_{i=1}^{2L^{a}+1}W_{i}\left[\chi_{i,k}^{x}-{\hat{x}}_{k|k-1}\right]{\left[\chi_{i,k}^{x}-{\hat{x}}_{k|k-1}\right]}^{T}$$

(33) $$P_{y,k}=\sum_{i=1}^{2L^{a}+1}W_{i}\left[\gamma_{i,k}-{\hat{y}}_{k}\right]{\left[\gamma_{i,k}-{\hat{y}}_{k}\right]}^{T}$$

(34) $$P_{xy,k}=\sum_{i=1}^{2L^{a}+1}W_{i}\left[\chi_{i,k}^{x}-{\hat{x}}_{k|k-1}\right]{\left[\gamma_{i,k}-{\hat{y}}_{k}\right]}^{T}$$

(35) $$K_{k}=P_{xy,k}P_{y,k}^{-1}$$

(36) $$P_{k|k}=P_{k|k-1}-K_{k}P_{y,k}K_{k}^{-1}$$

(37) $${\hat{x}}_{k|k}={\hat{x}}_{k|k-1}-K_{k}\left(y_{k}-{\hat{y}}_{k}\right)$$

Since the nonlinear system function is available in this paper, state estimation using UKF is easy to apply by giving the parameters of noise. To detect the fault, one can simply define the residual *e* as the difference between the system output and the estimated output and check if it exceeds a certain threshold.

To achieve the FTC using the proposed two-layer, multiple-model structure, a bank of unscented Kalman filters could be created to form a set of interval observers, which has the ability to isolate the fault and determine the faulty interval by checking the corresponding residuals.

(38) $$\left\{\begin{array}{l} {\hat{x}}_{k,i}=UKF\left(f_{\theta i},{\hat{x}}_{k-1},u_{k}\right)\\ {\hat{y}}_{k,i}=h\left({\hat{x}}_{k,i}\right) \end{array}\right.$$

(39) $$e_{k,i}=y_{k}-{\hat{y}}_{k,i}$$

where UKF denotes the unscented Kalman filter and $f_{\theta i}$ is a system function with the faulty parameter $\theta_{i}$.

One thing should be noticed is that the isolation of the fault should be carried out when the effect of the fault is getting relatively stable. Otherwise, the result given in the transient period may not be trustworthy. For this reason, one defines an index *z* which equals to the absolute value of the derivative of residuals to determine if it is the time to do interval checking.

(40) $$z_{i}≜\left|diff\left(e_{i}\right)\right|$$

The FDI strategy would be implemented first by checking elements of $z_{k,i}$ to see if at least one of them exceeds the detection threshold. If it holds, a fault is detected while it is happening. Next would be checking $z_{i}$ step by step when all of its elements are not higher than the fault isolation threshold, which means estimations are stable and it is time to determine the fault interval. At this moment, residuals $e_{k}$ would behave with interval features. It is easy to find the two filters who hold the zero residual by checking if $e_{k,i}\cdot e_{k,i+1}\leq 0$.

**Figure 7:**
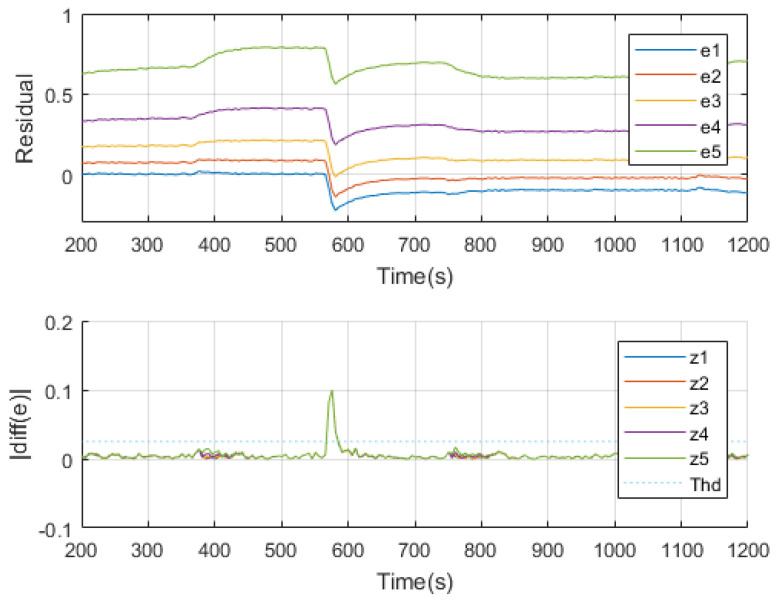
FDI process using interval unscented Kalman filters.

As is illustrated in Figure 7, a fault is introduced at 560 s. It is detected several seconds when one of $z_{i}$ beyond the threshold. After about 20 s, all of $z_{i}$ are reduced and below the threshold. At this moment, behaviors of the residuals become stable and it is easy to see that $e_{2}$ and $e_{3}$ cover the zero axis, indicating the fault value is in the assumed second interval. Thus the fault is isolated.

### 5.2. FTC Implementation with the Two-Layer, Multiple-Model Structure

**Figure 8:**
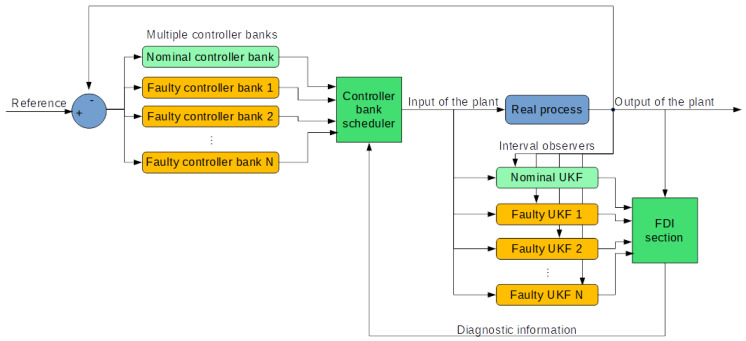
FTC strategy using two-layer, multiple-model structure.

After the preparation of the former sections, we have unified controller banks designed from homogeneous two-layer model banks. As the second layer model banks are concerned about faults, their corresponding controller banks have the ability to maintain system performance in particular faulty situations. The entire FTC strategy is shown in Figure 8.

In the strategy, FDI section would monitor the real process using estimations given by interval UKFs and generate diagnostic information to guide the controller bank scheduler. When a fault is detected and isolated, there are two possible situations: the fault is in the assumed intervals or the fault is beyond the edges of the intervals. The first situation is the majority cases according to our design purpose. Like the case in Figure 7, the fault is diagnosed between the second and the third intervals. It is determined from checking the indication term $e_{2}\cdot e_{3}$ that is less than zero, meaning the two values have different signs and thus cover the zero residual. To compensate the fault, the corresponding two controller banks are selected to give a weighted control output fro the faulty system. The weights are calculated using values of the residuals.

(41) $$\left\{\begin{array}{cc} w_{i} & =1-\frac{|e_{i}|}{|e_{i}\left|+\right|e_{i+1}|}\\ w_{i+1} & =1-\frac{|e_{i+1}|}{|e_{i}\left|+\right|e_{i+1}|} \end{array}\right.$$

(42) $$u=u_{i}w_{i}+u_{i+1}w_{i+1}$$

where *w* indicates the weight the controller bank, *i* is the index of the second layer controller bank and *u* denotes the control output.

One special case in this “within-interval” situation is the one when $e_{i}\cdot e_{i+1}=0$. It means the current system behaves exactly the same as one assumed faulty situation. Using (41) and (42) to calculate a new control signal is also valid because the corresponding weight will equal 1 to have suitable assignment.

For the “beyond-interval” situation, the term $e_{i}\cdot e_{i+1}\leq 0$ does not hold, meaning the faulty system behaves beyond the worst situation. It is very rare and one can only activate the controller bank of the closest assumed faulty case to compensate the fault to some extent.

### 5.3. Simulation Results with Faults Affecting Heat Transfer Coefficient

The FTC strategies described in the former sections are simulated here. Key information about the HEX reactor, exothermic reaction and initial states of the simulation are listed in Table 1. Besides, concentrations of sodium the two reactants are both set to 9% in mass just as the experiments did.

**Figure 9:**
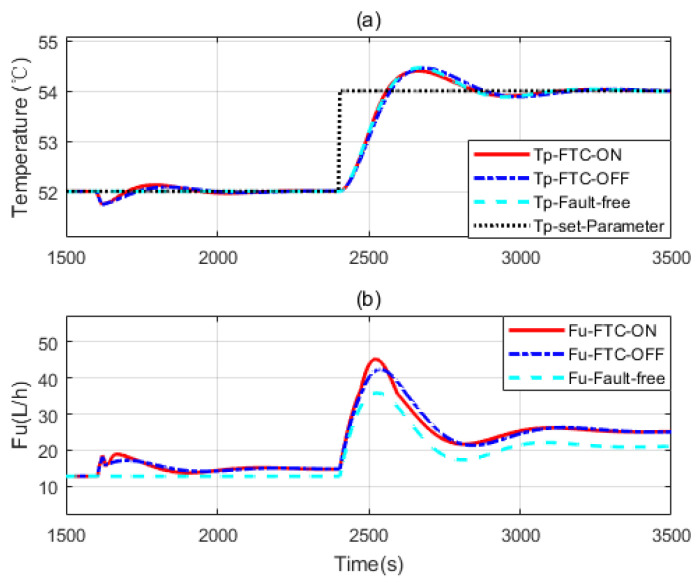
(**a**,**b**) The simulation considering a faulty parameter dropping to 65% of its nominal value.

**Figure 10:**
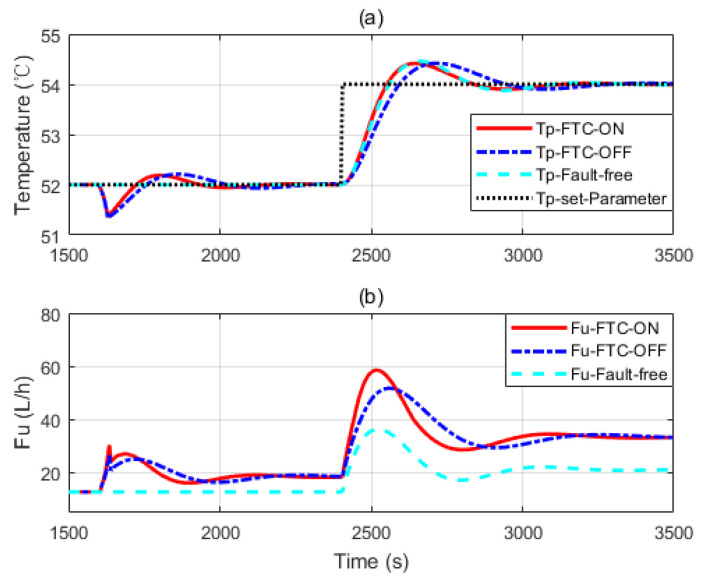
(**a**,**b**) The simulation considering a faulty parameter dropping to 45% of its nominal value.

Four simulations are presented here. In Figure 9 and Figure 10, faults are introduced at 1600 s. Heat transfer coefficient drops to 65% and 45% of its nominal value respectively. Control reference changes at 2400 s.

**Figure 11:**
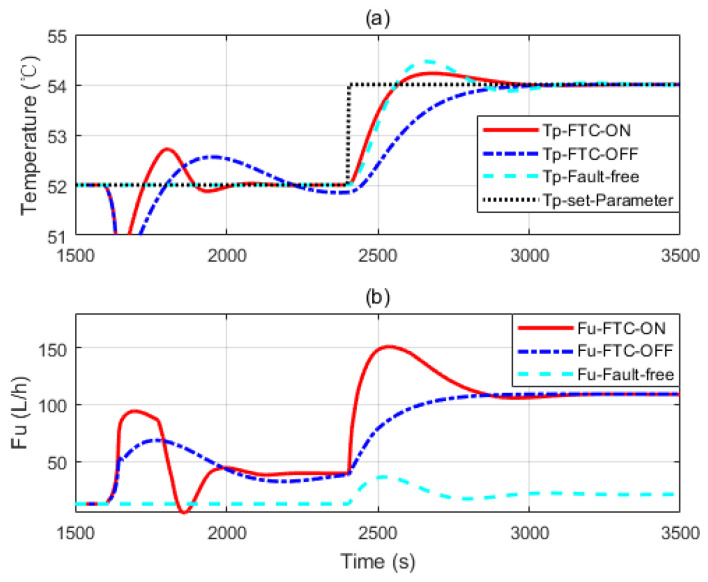
(**a**,**b**) The simulation considering a faulty parameter dropping to 18% of its nominal value.

Figure 9a and Figure 10a show three independent simulation outputs of the HEX reactor, FTC on, FTC off and fault free cases. Figure 9b and Figure 10b present corresponding control signals given by the controller banks. It can be seen from Figure 9 that when the fault occurs, no matter whether the FTC strategy is turned on or off, controller banks could bring the faulty system to the desired output. However, it performs slightly better when the FTC strategy is turned on. When the reference changed at 2400 s, all the controller banks reacted to that. The one in which FTC was turned on also behaved a little bit better than that when FTC was turned off. It was really close to the performance of the controller bank corresponding to the fault-free situation. That means the FTC strategy works and provides proper compensation to the faulty system. To demonstrate the effectiveness of the proposed design strategy, simulations with serious faults are presented. More obvious results are shown in Figure 10 and Figure 11. One thing that should be noticed is that the faulty values were chosen randomly. They were used to do simulations to show that the proposed design strategy should work as soon as the faulty range is covered by the two-layer, multiple-model structure.

From Figure 10 and Figure 11, we can see that more aggressive controls are given by the controller banks under the FTC strategy. It helps the faulty system to recover fast. Figure 11 belongs to the third case of the former section: the fault is severe and the faulty system behaves beyond the interval of the second layer model banks. Therefore, when the fault is detected, controller banks corresponding to the model bank with a preset-fault at 20%h are activated to handle the problem. Though it may not be the perfect FTC strategy, it is the optimal one under all the assumptions.

**Figure 12:**
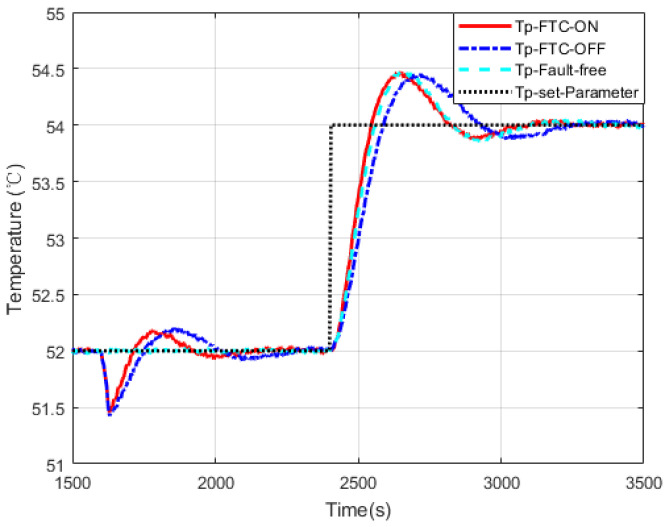
The simulation of a 45% fault case considering measurement noise.

Figure 12 presents a simulation result considering measurement noise for the case in Figure 10. It shows that the proposed FTC strategy also works well in a noisy situation. Other cases have the similar results under measurement noise.

### 5.4. Simulation Results with Faults Affecting the Temperature of Input Utility Fluid

Another simulation about the faults affecting the temperature of utility input was done the same way as in the former sub-section. According to previous assumptions, we measured only $T_{p}$ and manipulated only $F_{u}$ of the system. Other parameters were seen as constants. In this case, we considered that there was a fault, for instance, the failure of heater in utility source tank or the damage of the insulation material of that tank, which would affect the temperature of utility input.

In this simulation, we kept all the conditions as in Table 1. For the targeting parameter $T_{uin}$, besides its nominal value 59.4 °C, four faulty situations were set at 57.2 °C, 55.0 °C, 52.8 °C, 50.6 °C. A fault, the temperature of the utility input dropping from its nominal value to 93% of that value (55.242 °C), was introduced at 3200 s.

**Figure 13:**
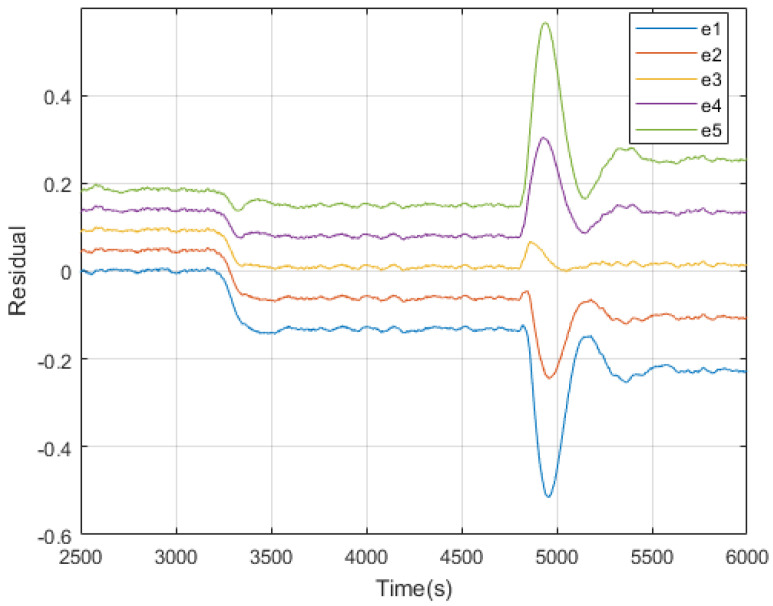
The simulation of interval residuals of $T_{uin}$ fault.

**Figure 14:**
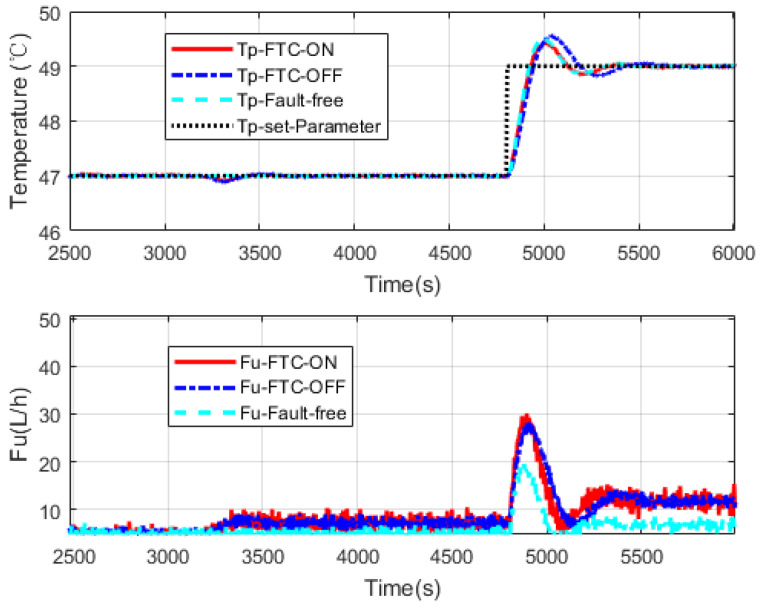
FTC simulation of $T_{uin}$ fault.

Simulation results are presented below. In Figure 13, interval residuals calculated from UKF estimations show us the state of the system at each time point. It is clear that before the fault occurs at 3200 s, residuals of UKF1 are around zero, which means the system is in a normal state. When the fault comes, all UKFs have reactions. After the transient period, the intervals become stable and it is easy to see that residuals corresponding to UKF2 and UKF3 cover the zero axis, indicating the fault is in this interval. One thing interesting is that there are big fluctuations around 5000 s. Though their magnitudes are much higher than the changes before, the interval covering the zero axis stays unchanged before and after. This is because they are not caused by a fault, but the controller effect from a change in the reference input; see Figure 14.

Like the cases in Figure 9 and Figure 10, the controller bank of the nominal model has the ability to maintain the system in a faulty situation to some extent. However, when the FTC strategy is applied, it switches to a suitable controller bank in faulty situations and presents better performance than the case when FTC strategy is turned off, which also illustrates the effectiveness of the proposed method.

## 6. Conclusions

In this paper, an intensified heat-exchanger/reactor is introduced and a fault tolerant control strategy using a two-layer, multiple-model structure is proposed for this system.

The HEX reactor points out a new direction for the development of classical batch reactors. However, its dynamics under chemical reactions are complex and of highly nonlinear. Traditional methods for its controller design are complicated and difficult. It is even more difficult when considering FTC applications. To handle this problem, a multiple-model approach and its divide-and-conquer strategy were used to construct a two-layer, multiple-model structure. Among this structure, the first layer considers a simple description of the nonlinear system and the second layer concerns faults. As the mathematical model is already available, virtual experiments could be done to generate enough IO data for the creation of multiple-model banks by using system identification method. Additionally, then, the model predictive control approach was used to design controllers by using the information of model banks. A switching strategy combines local models and local controllers to give out unified outputs of each model bank and controller bank respectively. The FDI section uses the unscented Kalman filter to estimate the states of the reactor and forms indexes to show the intervals of faults. For the FTC implementation, both switching and linear merging schemes are used according to the faulty situations. After the tuning of controller banks, the fault tolerant control of the HEX reactor was simulated in two kinds of faults. Simulation results proved the validity of the proposed FTC strategy. The complexity of handling the FTC design for the nonlinear systems is greatly reduced under the proposed method. However, an accurate nominal model of the system is still a pre-condition for applying it.

## Figures and Tables

**Table 1:** Key information about the simulation.

Notation	Description	Value
$M_{w}$	Mass of the HEX reactor	10.84 kg
$A_{p}$	Heat exchange area of process channel	$2.68\times 10^{4}$ mm^2^
$A_{u}$	Heat exchange area of utility channel	$4.56\times 10^{5}$ mm^2^
$E_{a}$	The activation energy	$7.61\times 10^{4}$ J· mol^−1^
$k_{j}^{0}$	Pre-exponential factor of the reaction	$8.13\times 10^{11}$ L· mol^−1^· s^−1^
$F_{p1}$	Flow-rate of ${\mathrm{Na}}_{2}S_{2}O_{3}$	4.7 L · h^−1^
$F_{p2}$	Flow-rate of $H_{2}O_{2}$	2.3 L · h^−1^
$T_{pin}$	The input temperature of process fluid	21.1 °C
$T_{uin}$	The input temperature of utility fluid	59.4 °C

## Abbreviations

The following abbreviations are used in this manuscript:

HEX reactor	Heat-exchanger/reactor
FTC	Fault tolerant control
EKF	Extended Kalman filter
UKF	Unscented Kalman filter
FDI	Fault detection and isolation
FDD	fault detection and diagnosis
MPC	Model predictive control
ARX	Auto regressive exogenous
IO	Input and output
SISO	Single input single output

## References

[1] Etchells J.C. (2005). Process Intensification: Safety Pros and Cons. Process Saf. Environ. Prot..

[2] Green A., Johnson B., John A. (1999). Process intensification magnifies profits. Chem. Eng..

[3] Hendershot D.C. (2000). Process minimization: Making plants safer. Chem. Eng. Progress..

[4] Anxionnaz Z. (2008). Heat exchanger/reactors (HEX reactors). Chem. Eng. Process..

[5] Benaissa W., Elgue S., Gabas N., Cabassud M., Carson D., Demissy M. (2008). Dynamic Behaviour of a Continuous Heat Exchanger/Reactor after Flow Failure. Int. J. Chem. React. Eng..

[6] Mogens B., Michel K., Jan L., Marcel S. (2006). Diagnosis and Fault-Tolerant Control.

[7] Yang H., Jiang B., Staroswiecki M. (2009). Supervisory fault tolerant control for a class of uncertain nonlinear systems. Automatica.

[8] Li Z., Dahhou B., Li Q., Zhang M. (2015). Design of passive fault tolerant control of a process system. Proceedings of the 27th Chinese Control and Decision Conference (2015 CCDC).

[9] Yu X., Zhang Y. (2015). Design of passive fault-tolerant flight controller against actuator failures. Chin. J. Aeronaut..

[10] Rotondo D., Nejjari F., Puig V. Passive and active FTC comparison for polytopic LPV systems. Proceedings of the 2013 European Control Conference (ECC).

[11] Jiang J., Yu X. (2012). Fault-tolerant control systems: A comparative study between active and passive approaches. Annu. Rev. Control.

[12] Youmin Z., Jin J. (2001). Integrated active fault-tolerant control using IMM approach. IEEE Trans. Aerosp. Electron. Syst..

[13] Magill D. (1965). Optimal adaptive estimation of sampled stochastic processes. IEEE Trans. Autom. Control.

[14] Murray-Smith R., Johansen T.A. (1997). Multiple Model Approaches to Modelling and Control.

[15] Dougherty D., Cooper D. (2003). A practical multiple model adaptive strategy for single-loop MPC. Control Eng. Pract..

[16] Murphey T.D. (2008). On multiple model control for multiple contact systems. Automatica.

[17] Ben Chabane S., Maniu C.S., Camacho E.F., Alamo T., Dumur D. Fault tolerant control approach based on multiple models and set-membership state estimation. Proceedings of the 2016 European Control Conference (ECC).

[18] Mirzaee A., Salahshoor K. (2012). Fault diagnosis and accommodation of nonlinear systems based on multiple-model adaptive unscented Kalman filter and switched MPC and H-infinity loop-shaping controller. J. Process Control.

[19] Théron F., Anxionnaz-Minvielle Z., Cabassud M., Gourdon C., Tochon P. (2014). Characterization of the performances of an innovative heat-exchanger/reactor. Chem. Eng. Process. Process Intensif..

[20] He M., Li Z., Han X., Cabassud M., Dahhou B. (2019). Development of a Numerical Model for a Compact Intensified Heat-Exchanger/Reactor. Processes.

[21] Westerterp K.R., Van Swaaij W.P.M., Beenackers A.A.C.M., Kramers H. (1991). Chemical Reactor Design and Operation.

[22] Orjuela R., Marx B., Ragot J., Maquin D. (2013). Nonlinear system identification using heterogeneous multiple models. Int. J. Appl. Math. Comput. Sci..

[23] Boukhris A., Mourot G., Ragot J. (2010). Non-linear dynamic system identification: A multi-model approach. Int. J. Control.

[24] Wang L., Wang L. (2009). Discrete-time MPC for Beginners. Model Predictive Control System Design and Implementation Using MATLAB^®^.

[25] Wu X., Xie Z., Bai X., Kwan T. (2019). Design of a 1-bit MEMS Gyroscope using the Model Predictive Control Approach. Sensors.

[26] Julier S.J., Uhlmann J.K. A New extension of the Kalman filter to nonlinear systems. Proceedings of the Signal Processing, Sensor Fusion, and Target Recognition VI.

[27] Sayed W.E., Abd El Geliel M., Lotfy A. (2020). Fault Diagnosis of PMSG Stator Inter-Turn Fault Using Extended Kalman Filter and Unscented Kalman Filter. Energies.

